# The Week's Work

**Published:** 1910-03-19

**Authors:** 


### The Week's Work.

### BROWNLOW HILL WORKHOUSE.

The Liverpool Select Vestry, who are the masters of Brownlow Hill Poor Law Infirmary, decided in effect by their votes at a meeting on the 8th instant against making this famous infirmary an up-to-date modern hospital of the best type. Instead of rebuilding, they have decided to erect two additional sanitary towers in connection with the men's hospital—one for the acute wards and one for the infirm wards; to enlarge the central sanitary tower on the women's side; to reconstruct the whole of the kitchen, scullery, and bath accommodation of the lying-in wards; to provide a lift in connection with both men's and women's hospitals, and to make a few more minor changes. The ground on which this tinkering proposal was carried by a large majority was that the transitional state of the Poor Law rendered any more drastic and thorough building operations impracticable. Seeing that the expenditure involved was stated to be about £16,000, it seems to us that the Vestry has failed in its duty to the ratepayers by not waiting to see what may result from the report and recommendations of the Poor Law Commission, which they seem to put forward as a reason for not rebuilding the whole of this out-of-date and insanitary sick-house. No attempt has been made to answer the criticisms of Sir Henry Burdett published in The Hospital of January 1, and knowing that those criticisms only related to a relatively small portion of the whole establishment, it is fair to infer that if all the facts were to be published matters might prove to be infinitely worse than they were shown to be in the report in question. However, the action of the Liverpool Select Vestry in the special circumstances may be accepted as striking evidence of the desirability of abolishing Poor Law Guardians and placing the management of the whole service for the sick of this country in more responsible hands.

### THE POOR-LAW COMMISSION'S REPORT.

It is interesting to place on record the fact that 10,000 copies of the Blue Book containing the Majority and Minority Reports for England and Wales were printed, of which 6,820 copies have been sold. These reports were subsequently reprinted in a more convenient form, the Majority Report in two volumes and the Minority Report in one volume. Six thousand copies of each of these volumes were issued, and up to the present time only 1,300 copies of the Majority and 1,630 copies of the Minority report have been sold. The Poor Law Report for Ireland has excited infinitely less interest, for although 5,000 copies of it were printed only 450 copies have been sold. No doubt the sale of the Minority Report in a single volume has been materially diminished by the issue by Mr. and Mrs. Sidney Webb of their book on "English Poor Law Policy," which has had, we believe, a very large sale. A very handy and useful reprint of the material portions of both Reports was issued by the *Times*, which deserved, and we hope has attained to, a very extensive sale. This latter publication is one which we recommend to all our readers, because it is essential, if the new policy which is likely to arise on the issue of these reports is to be fruitful in results, that every intelligent resident in the British Isles should study the existing Poor Law system and understand its main principles, so as to secure that all changes, however radical they may be, shall be wisely conceived, and that care shall be taken that any authority substituted for the existing Guardians shall be efficient and representative.

### GENERAL HOSPITALS AND PAYING PATIENTS.

The Colonial Secretary in the Transvaal is engaged at the present time in endeavouring to do away with the anomaly under which patients in the Transvaal hospitals either get everything for nothing or have to pay full fees for everything. The practice in Pretoria, which may be taken as a good example of the existing system, is to charge a hospital patient so much a day, inclusive of medical attendance. In the Cape and some Transvaal hospitals a general practice has grown up under which patients pay for their medical attendant as well as the hospital fee. This latter system has the objection that many patients who are able and willing to pay 10s. a day are not in a position to pay in addition the practitioner's fees. At the present time, unless a patient can pay 10s. per diem, plus the medical fees, he is passed on to a pavilion where he is admitted either as a free patient or in some cases is permitted to pay for his maintenance alone. The present movement is to secure the adoption of a general system whereby a patient whose social position is known may secure the right to be admitted as an in-patient for an inclusive payment of 10s. per diem. The general adoption of this system would give great satisfaction to a very large class of persons who in health earn a fair and even a comfortable living, but who, by failure to make any provision against sickness, find great difficulty in paying for their treatment when disabled by disease or injury. The medical committees of the various hospitals are opposed to the proposal that they should give their services to patients paying 10s. a day, though that sum may prove remunerative to the hospital. The solution of the difficulty will probably be found in a system by which the whole of the patients' payments are pooled, and a percentage of them set aside for the remuneration of the medical practitioner in attendance upon each paying patient. British general hospitals are face to face with practically the same position in regard to in-patients at the present time, and the sooner the question is faced, threshed out, and settled upon a basis which is fair to the profession, the hospitals and the patients who desire to pay for their maintenance, the better. We indicated a plan upon which the difficulties to be faced may readily be met in The Hospital of February 5, page 547.

### HOSPITAL POPULATION.

In what manner is it possible to estimate the "hospital population" of any community? To those that have not gone fully into the question of hospital statistics it may seem strange that there can possibly be any doubt about the reliability of the ordinarily accepted methods. By "hospital population" is, of course, meant the number of individuals in a community likely to use the beds, or, in other words, likely to stand in need of institutional treatment. Farr's original computation was that to one annual death two individuals in the community are on an average constantly suffering from sickness severe enough to demand hospital treatment. This is easily understood. It means that in a city community the daily percentage of sick is double the annual percentage of deaths. The theory, however, has not always been proved true, and certainly is not absolutely true for rural communities. One of the latest and most searching investigations into hospital population statistics is the one recently completed by the New York Board of Health, which has resulted in showing that the Farr method is on the whole very accurate. Taking a very wide series of figures, it was found that the sick population of the community of 4,000,000 was 135,000, giving a daily percentage of 1.83, as against the old estimate of 3.3 which has been attributed to New York. This is the number of absolute sick, of whom the "hospital population" is probably 8 to 10 per cent. only. The old estimate is the one on which hospital workers have so far estimated their number of beds, and, as a writer has recently pointed out in one of the American magazines, there is a danger that New York may within the next ten years possess far more beds than are necessary for the hospital population. This statement is probably an exaggeration, though in any case it is highly improbable that the ratio of disease to population will remain stationary. The term "hospital population" is a bad one, in so far as it does not refer to that section of the community that is in want of hospital treatment—as it is understood in London, for instance—but simply to the section that is in need of clinical treatment. In New York, where paying patients are largely received in the hospitals, the theoretical disadvantages of a superfluity of hospital beds are much less obvious than they would be in Edinburgh, for example, or in London.

### THE HONORARY MEDICAL STAFF.

A question which is of general interest was raised at the annual meeting of the Royal Berkshire Hospital in reference to the position and duties of the physicians and so-called assistant-physicians of this institution. According to the Archdeacon of Berkshire and the minute of a sub-committee of January 1909, a change in the rules made in March 1904 has proved detrimental to the status of the physicians and has diminished their value both to the institution and to the public. This change was made at the time it was decided to appoint two general practitioners as assistant-physicians to the hospital without requiring them to be medically qualified as physicians under the rules. These assistant-physicians were each granted a certain number of beds. The physicians, or some of them, claim that they have been injured by losing a certain number of beds, and by the circumstance that the assistant-physicians have been placed in a more favourable position than the physicians as regards their professional work both inside and outside the hospital. The assistant-physicians not only attend all the out-patients but have beds of right, and practise as physicians under rule 54, and they are free from the restrictions imposed upon the physicians, too. These so-called assistant-physicians are at liberty to practise pharmacy and surgery, which, of course, the physicians are restrained from doing. In brief the grievance is that the assistant-physicians at the present time combine in their office the privileges of physicians to the hospital, to the injury of their seniors, with the advantages and emoluments of general practitioners outside. Unless this state of affairs is altered it is maintained with some show of reason that no member of the profession possessing the qualifications required under the rules for a physician will be likely to apply in future for the appointment of physician to the Royal Berkshire Hospital. The speakers who opposed the Archdeacon's resolution for an inquiry by the Royal College of Physicians included Sir James Clark, who admitted that the physicians had lost status in consequence of the change in the rules, and maintained it to be probable that such loss would never be recovered, because in a town like Reading, so near London, the hospital would not be able to get a consulting medical staff. He further maintained that the titles physician and assistant-physician of a hospital were purely courtesy titles, and did not imply that their holders must be members or Fellows of the Royal College of Physicians. On this point Sir James Clark is clearly wrong, and it seems to us a great pity that, instead of voting down the Archdeacon, the governors did not agree to call in an independent and high authority like that suggested to go into the whole question at issue in private conference, and so to secure an amicable adjustment of differences which, if continued, cannot fail to injure the reputation and popularity of the Royal Berkshire Hospital.

### THE DRUG MARKET.

Another sharp advance in the value of ipecacuanha is the most important price alteration of the week, and quotations are now about double the price at which ipecacuanha could be bought at the beginning of the year. Supplies are very scarce, and it is not unlikely that prices will advance to a still higher figure; preparations of this drug are dearer in proportion. Buchu leaves are again cheaper, and there is some probability that before long normal values will be reached. Quotations for cod-liver oil are inclined to become lower. Oil of cloves is slightly dearer, owing to an advance in the price of cloves. Coca leaves are somewhat higher in price, and balsam tolu is a fraction dearer. Prices asked for saffron are appreciably higher.

